# Supplementary material for: PBK/TOPK mediates Ikaros, Aiolos and CTCF displacement from mitotic chromosomes and alters chromatin accessibility at selected C2H2-zinc finger protein binding sites
Source: Nat Commun. 2025 Sep 23;16:8348. doi: 10.1038/s41467-025-63740-4 (PMC12457614; doi:10.1038/s41467-025-63740-4)
Supplement: Supplementary file 1 — Supplementary Information [file 41467_2025_63740_MOESM1_ESM.pdf]

Supplementary Information

PBK/TOPK mediates Ikaros, Aiolos and CTCF displacement from mitotic chromosomes and alters chromatin accessibility at selected C2H2-zinc finger protein binding sites

Dimond, A. *et al.*

Supplementary Figures 1-6

Supplementary Tables 1-5

Supplementary References

Uncropped blots for Supplementary Figs. 4c and 4f

Supplementary Figure 1

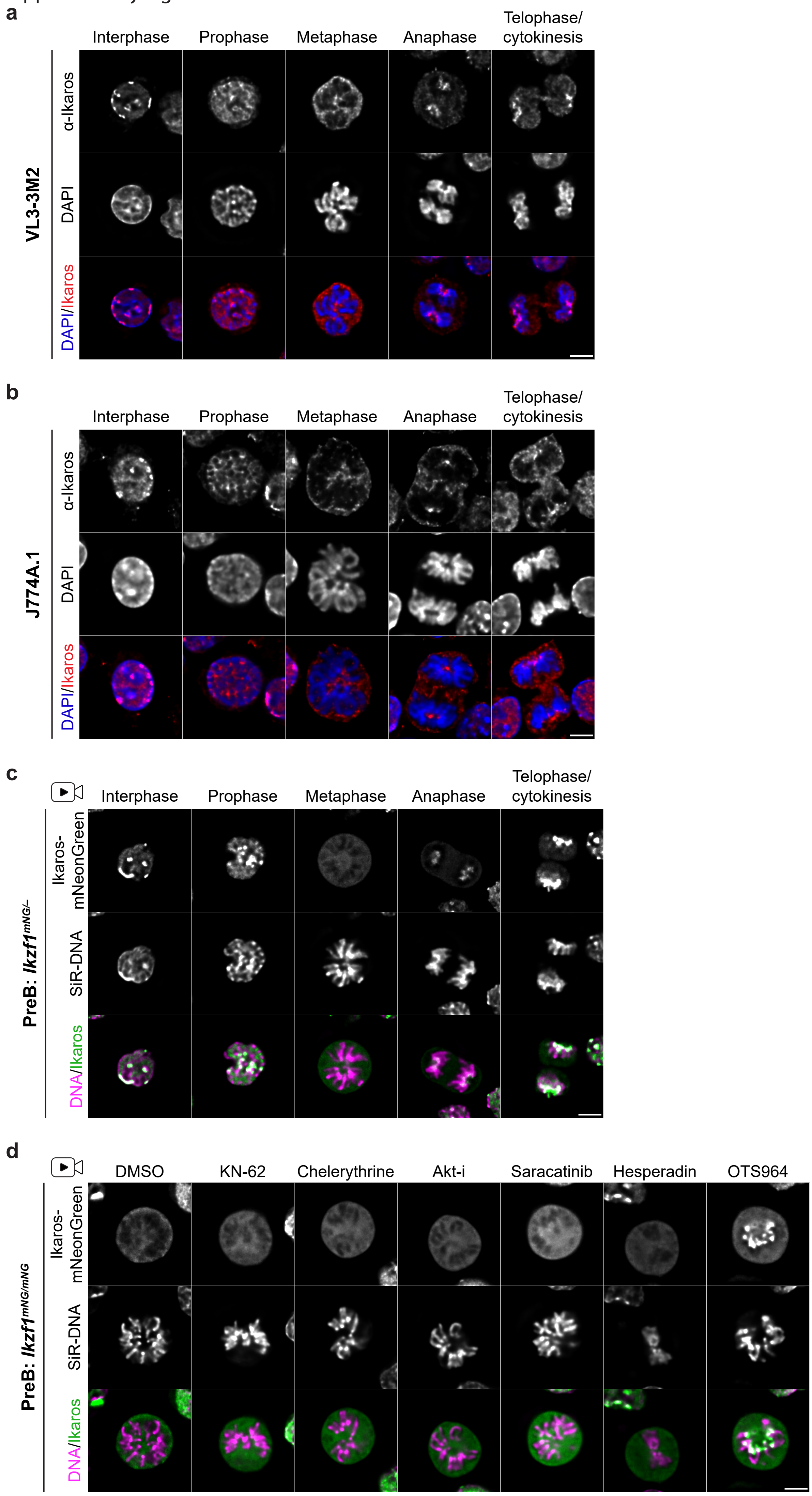

Supplementary Figure 1

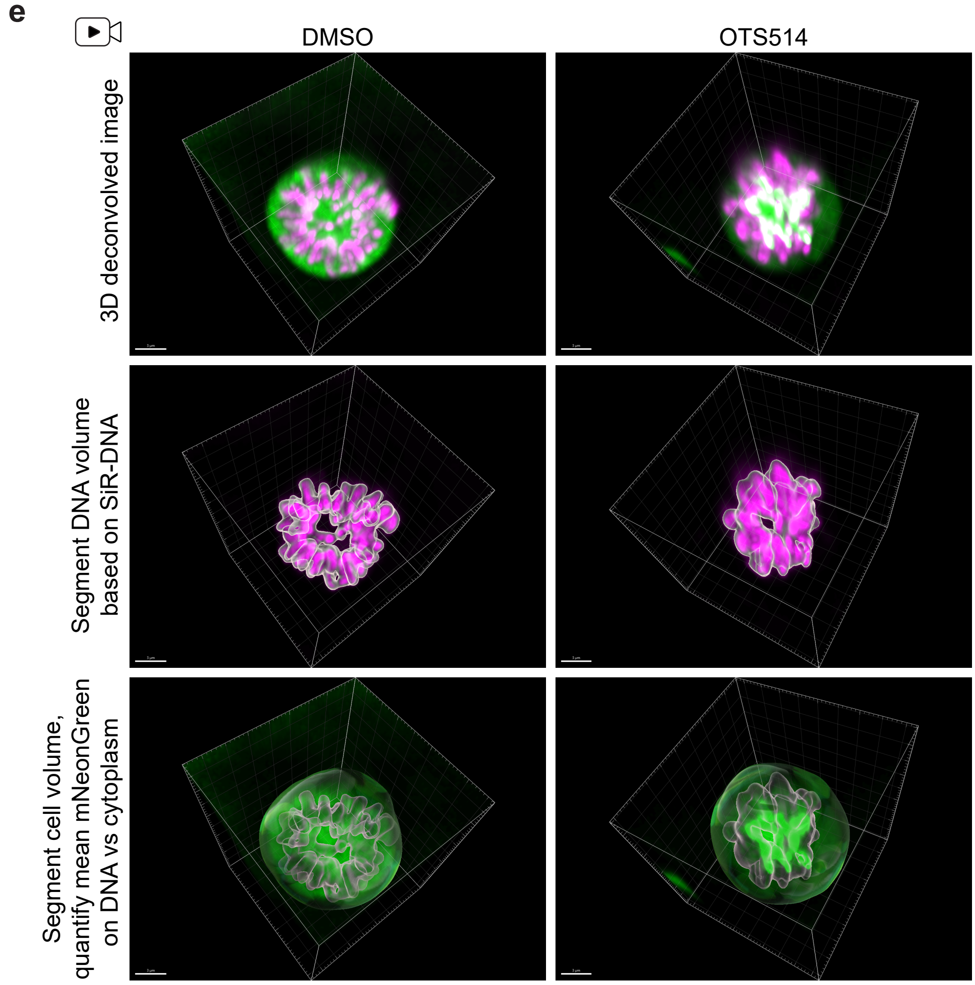

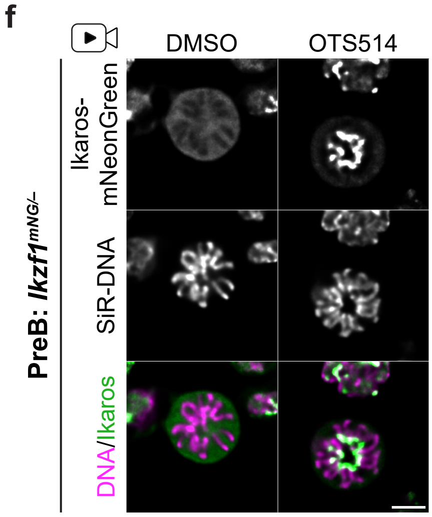

Supplementary Figure 1 | Ikaros dissociates from metaphase chromosomes in lymphoid and myeloid lineages, but is retained following specific kinase inhibition.

a. Immunofluorescence staining of Ikaros localisation in interphase and through mitosis in fixed VL3-3M2 cells (mouse T cell line) from asynchronously dividing cultures. Images are representative of at least seven cells per mitotic stage (>20 for metaphase) across two independent staining experiments. Scale bar=5 µm.

b. Immunofluorescence staining of Ikaros localisation in interphase and through mitosis in fixed J774A.1 cells (mouse macrophage cell line) from asynchronously dividing cultures. Images are representative of at least five cells per mitotic stage (>15 for metaphase) across two independent staining experiments. Scale bar=5 µm.

c. Live-cell images of Ikaros-mNeonGreen in interphase and through mitosis in $\textit{Ikzf1}^{mNeonGreen/–}$ heterozygous KI asynchronously dividing mouse preB cells (clone 1.2 from Fig. 1b) cultured with SiR-DNA. Images are representative of at least six cells per mitotic stage (>20 for metaphase), collected across three independent imaging experiments. Scale bar=5 µm.

d. Live-cell images of $\textit{Ikzf1}^{mNG/mNG}$ mitotic KI mouse preB cells (clone 2.1) from asynchronously dividing cultures showing Ikaros-mNeonGreen localisation following 10 min treatment with 10 µM of the indicated inhibitors. Cells were pre-cultured with SiR-DNA; scale bar=5 µm. Images are representative of at least two independent treatment experiments, with a minimum of 12 mitotic cells imaged per treatment in total.

e. Strategy for quantifying mean chromosomal and cytoplasmic Ikaros-mNeonGreen signal, shown for representative DMSO and OTS514-treated $\textit{Ikzf1}^{mNG/mNG}$ mitotic mouse preB cells. Cropped and deconvolved z-stack images were opened in Imaris software (upper). SiR-DNA signal was used to segment chromosomes (middle) and total cell volume was segmented by background mNeonGreen signal (bottom). Segmented volumes were used to construct a cell in Imaris software, allowing chromosomal and cytoplasmic mNeonGreen mean intensities to be measured separately and expressed as a ratio. Scale bars=3 µm.

f. Representative live-cell images of mitotic $\textit{Ikzf1}^{mNG/–}$ mouse preB cells (clone 1.2) from asynchronously dividing cultures following 10 min treatment with DMSO or 10 µM OTS514. Cells were pre-cultured with SiR-DNA; scale bar=5 µm. Images are representative of >25 mitotic cells from across two independent treatment experiments.

Supplementary Figure 2

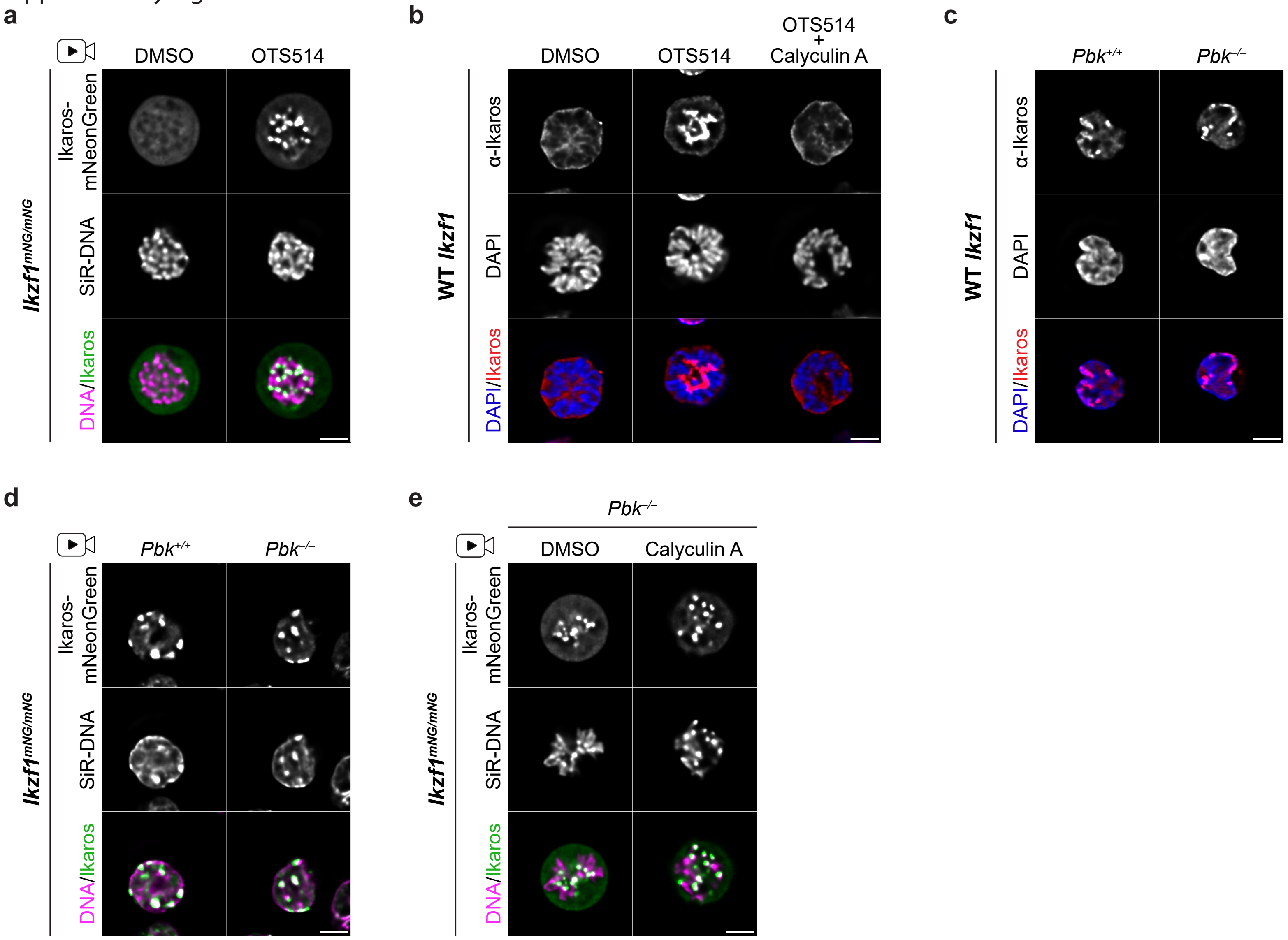

Supplementary Figure 2 | PBK activity is required for Ikaros dissociation in mitosis.

a. Representative live-cell images of *Ikzf1*$^{mNG/mNG}$ mouse preB cells which were arrested with demecolcine for 5 h, followed by 10 min treatment with DMSO or 10 µM OTS514. Images are representative of two independent experiments. Scale bar=5 µm.

b. Immunofluorescence staining of untagged Ikaros localisation in fixed mitotic preB cells following 10 min treatment of asynchronously diving cultures with DMSO or with 10 µM OTS514 alone or in combination with 100 nM Calyculin A. Images are representative of >18 treated mitotic cells from two independent experiments. Scale bar=5 µm.

c. Immunofluorescence staining of untagged Ikaros showing association with heterochromatin foci in both *Pbk*$^{+/+}$ and *Pbk*$^{-/-}$ interphase mouse preB cells. Images are representative of interphase cells from three independent staining experiments. Scale bar=5 µm.

d. Live-cell imaging of *Pbk*$^{+/+}$ and *Pbk*$^{-/-}$ interphase mouse *Ikzf1*$^{mNG/mNG}$ preB cells cultured with SiR-DNA. Images are representative of interphase cells from three independent imaging experiments. Scale bar=5 µm.

e. Live-cell imaging of *Ikzf1*$^{mNG/mNG}$ *Pbk*$^{-/-}$ mitotic mouse preB cells from asynchronously dividing cultures treated for 10 min with DMSO or 100 nM Calyculin A. Cells were pre-cultured with SiR-DNA; scale bar=5 µm. Images are representative of four independent replicates. All Calyculin A treated mitotic cells imaged (36/36) showed clear foci of mNeonGreen signal at centromeres, despite visible loss of chromosome spindle attachment in many cells.

Supplementary Figure 3

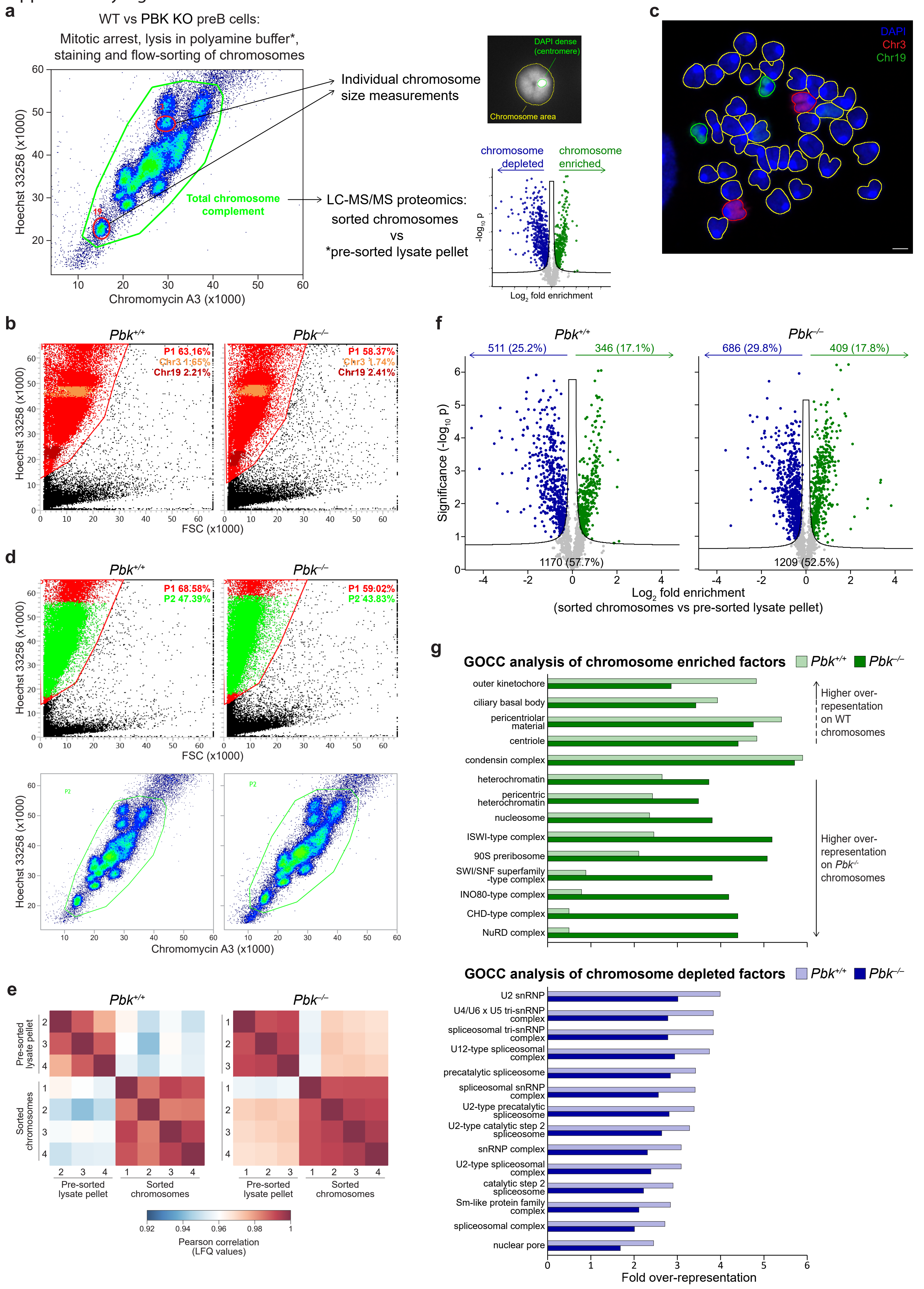

Supplementary Figure 3 | Analysis of mitotic chromosome size and protein composition in *Pbk*$^{+/+}$ and *Pbk*$^{-/-}$ mouse preB cells.

a. Strategy for purifying individual (red) or total (green) native mitotic chromosomes by flow-cytometry for size measurements or LC-MS/MS proteomics analysis respectively. Mitotically arrested cells were lysed; chromosomes were released into polyamine buffer and stained with Hoechst 33258 and Chromomycin A3 for visualisation/purification by flow cytometry. For size measurements, individual chromosomes 3 and 19 were isolated, cytospun onto poly-L-lysine slides and stained with DAPI, allowing total and DAPI-dense (centromeric) areas to be measured. For proteomics, total purified chromosomes were analysed by LC-MS/MS and compared to unpurified mitotic lysate pellets to calculate chromosomal enrichment or depletion of factors.

b. P1 gating strategy for isolation of individual chromosomes 3 and 19. Gates and percentages of total events are shown for a representative experiment, corresponding to Fig. 3a.

c. Representative metaphase spread from arrested mouse preB cells (example is from *Pbk*$^{-/-}$ cells) hybridised with paints/probes for mouse chromosomes 3 (red) and 19 (green). Chromosome outlines were segmented on DAPI signal (blue) and filtered based on chromosome paint signals. Scale bar=2 µm.

d. Gating strategy for flow-sorting total chromosomes. Gates and percentages of total events are shown for a representative experiment.

e. Pearson's correlations of proteomics samples after LFQ normalization, showing clear segregation of pre-sorted and sorted samples from both of *Pbk*$^{+/+}$ and *Pbk*$^{-/-}$ cells (rather than by replicate). *Pbk*$^{+/+}$ pre-sorted lysate pellet replicate 1 and *Pbk*$^{-/-}$ pre-sorted lysate pellet replicate 4 were excluded for technical reasons (see Methods).

f. Volcano plots of factors enriched (green) or depleted (blue) from *Pbk*$^{+/+}$ (left) and *Pbk*$^{-/-}$ (right) mitotic chromosomes, compared to pre-sorted lysate pellets (modified two-tailed *t*-test with permutation-based false discovery rate (FDR)<0.05 and S0=0.1; n=4 chromosome samples and n=3 lysate pellet samples).

g. GO term (cellular component) overrepresentation analysis amongst factors enriched (upper, green) or depleted (lower, blue) on mitotic chromosomes. Analysis was performed separately for *Pbk*$^{+/+}$ (pale green/blue) or *Pbk*$^{-/-}$ (solid green/blue) data, using the total proteins detected in each condition as background. Displayed are the topmost overrepresented terms with FDR<0.05, in either condition, ordered by the difference in fold overrepresentation between conditions.

Supplementary Figure 4

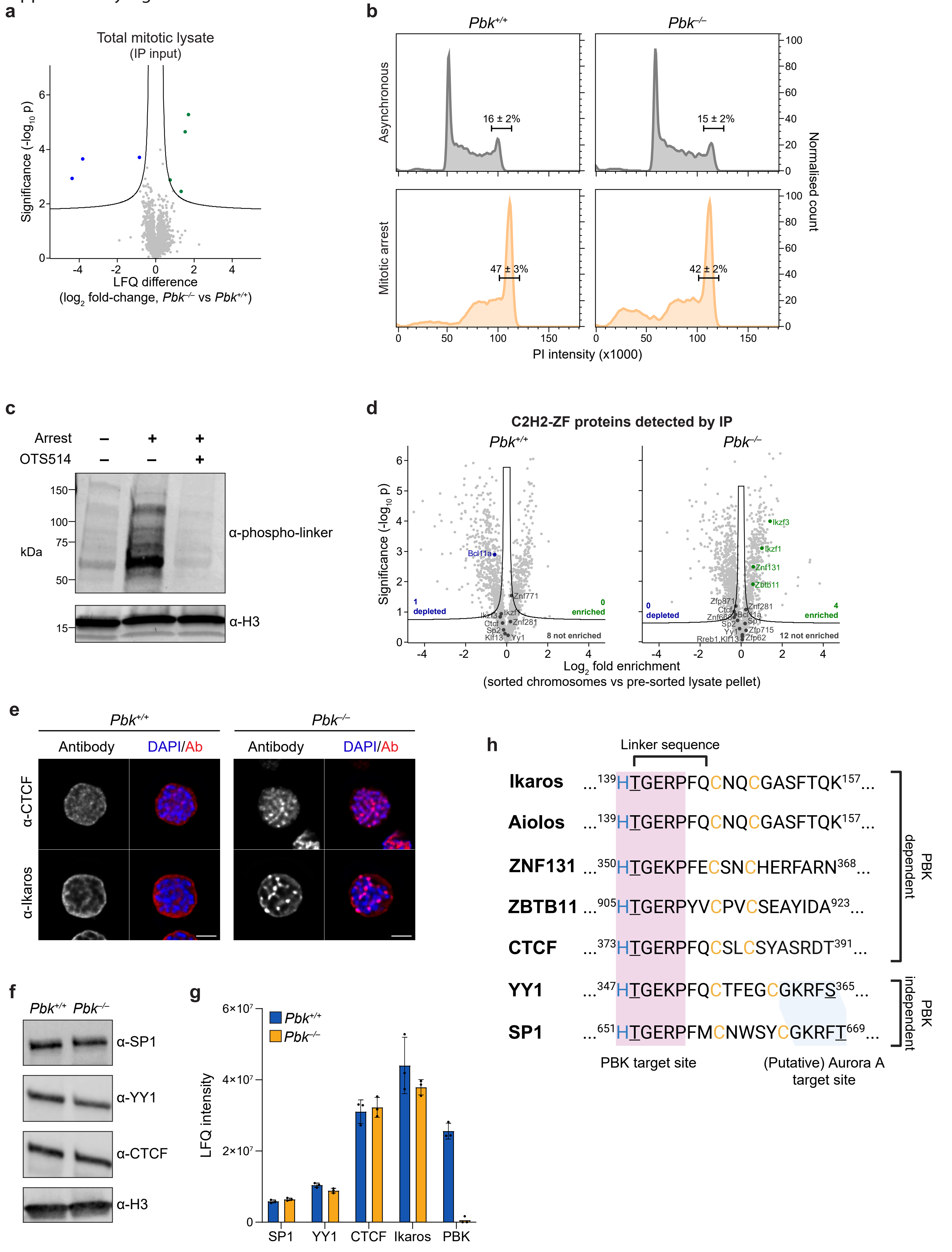

Supplementary Figure 4

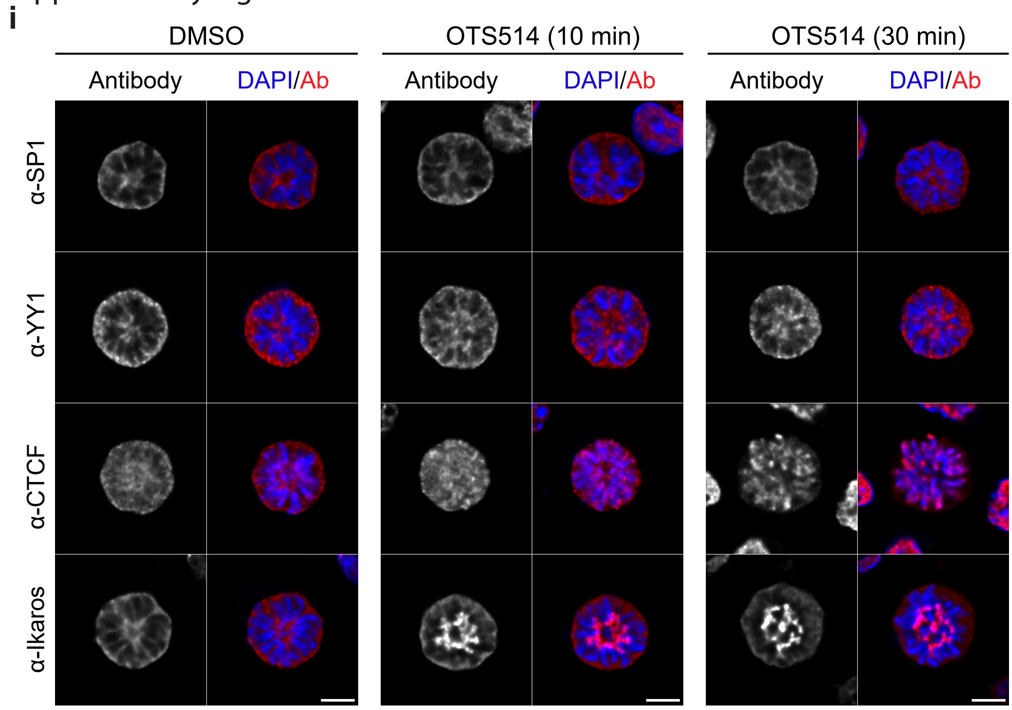

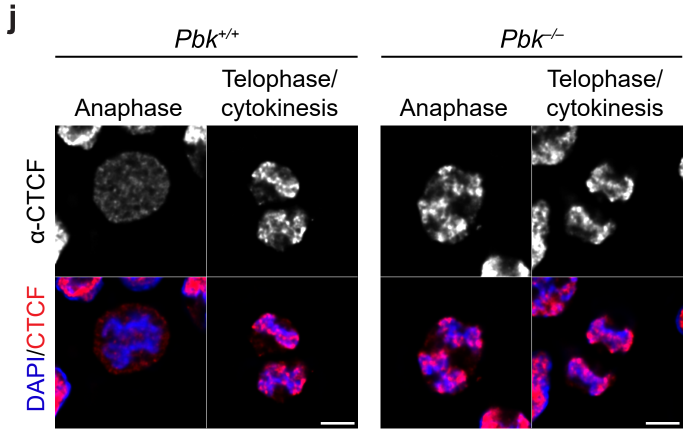

Supplementary Figure 4 | Loss of PBK activity alters mitotic phosphorylation status and localisation of C2H2-ZF proteins rather than abundance.

a. Volcano plot comparing protein abundances (LC-MS/MS) in total mitotic lysates from *Pbk*$^{+/+}$ and *Pbk*$^{-/-}$ mouse preB cells (Fig. 4c inputs). Only seven proteins differ significantly (green/blue; modified two-tailed *t*-test with permutation-based FDR<0.05, S0=0.1; *n*=3); two factors (including PBK) were robustly detected only in *Pbk*$^{+/+}$ and eight only in *Pbk*$^{-/-}$ (none are C2H2-ZF proteins).

b. Propidium iodide (PI) profiles of asynchronous (grey) and mitotically arrested (orange) *Pbk*$^{+/+}$ and *Pbk*$^{-/-}$ mouse preB cells. Mean G2/M percentages (±SD) from three replicates.

c. Western blot of mitotically arrested WT mouse preB cells, showing OTS514-induced loss of phospho-linker signal (10 µM, 10 min). Anti-H3=loading control; representative of two replicates.

d. Volcano plots of factors enriched/depleted from *Pbk*$^{+/+}$ and *Pbk*$^{-/-}$ mitotic chromosomes (as in Fig. 3e) highlighting PBK-phosphorylated C2H2-ZF proteins from Fig. 4c (green=enriched, blue=depleted, dark grey=not significant; modified two-tailed *t*-test with permutation-based FDR<0.05, S0=0.1; chromosomes *n*=4, lysate pellets *n*=3).

e. CTCF and Ikaros staining in fixed *Pbk*$^{+/+}$ and *Pbk*$^{-/-}$ mitotically arrested mouse preB cells. Representative of two experiments. Scale bar=5 µm.

f. Western blot of SP1, YY1 and CTCF in *Pbk*$^{+/+}$ and *Pbk*$^{-/-}$ mouse preB cells. Representative of two replicates; validated in another *Pbk*$^{-/-}$ clone. Anti-H3=loading control.

g. Abundance of selected factors in *Pbk*$^{+/+}$ and *Pbk*$^{-/-}$ mouse preB mitotic lysates (data/statistics from Supplementary Fig. 4a; bars=mean±SD). PBK detected only in *Pbk*$^{+/+}$, others not significantly different.

h. Sequence comparisons of selected C2H2-ZF proteins showing PBK-dependent or PBK-independent mitotic dissociation. Known/putative PBK and Aurora kinase A phosphorylation target sites are highlighted. Aurora kinase A phosphorylates YY1 at serine 365[19]. A similar motif is present in SP1 but absent from the others.

i. Immunofluorescence of the specified factors in mitotic mouse preB cells (from fixed asynchronous cultures), treated with DMSO/OTS514 (10 µM) for 10-30 min. Representative of ≥two independent experiments. Scale bar=5 µm.

j. CTCF staining in anaphase and telophase/cytokinesis *Pbk*$^{+/+}$ or *Pbk*$^{-/-}$ preB cells (from fixed asynchronous cultures). Representative of three experiments. Scale bar=5 µm.

Source data for Supplementary Figs. 4b-c and 4f-g are provided as a Source Data file or at the end of this file.

Supplementary Figure 5

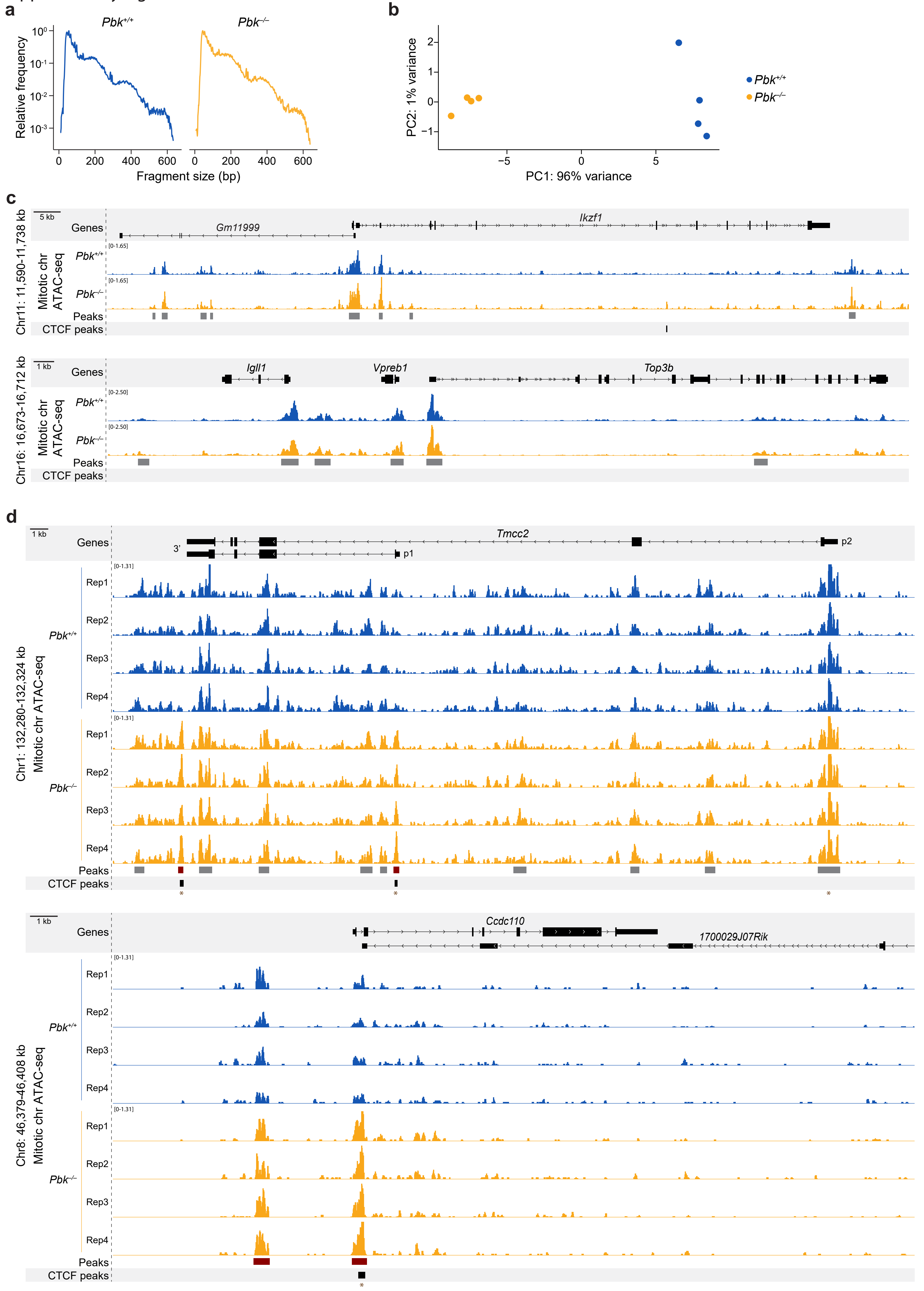

Supplementary Figure 5

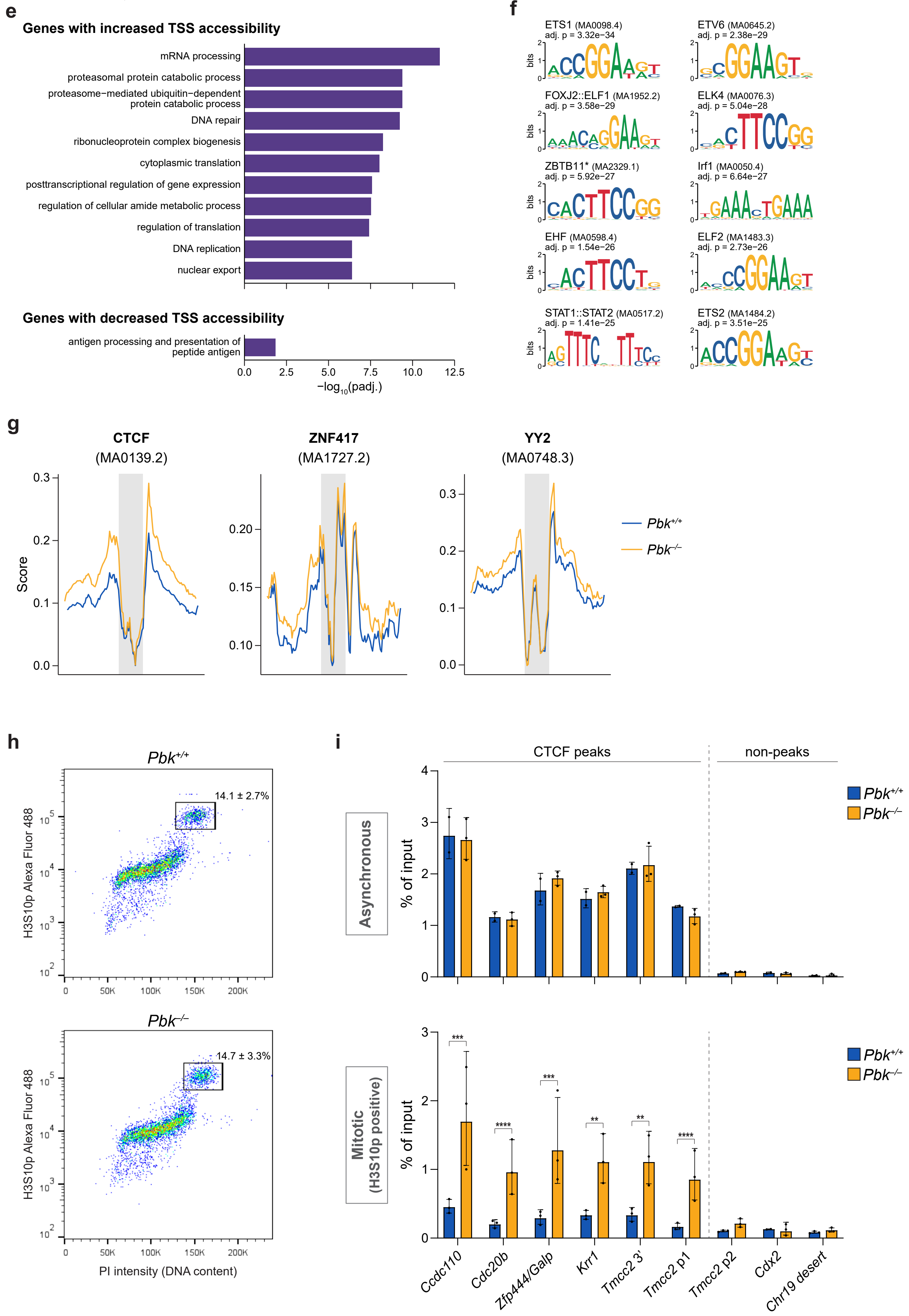

Supplementary Figure 5 | Chromatin analyses of purified mitotic chromosomes from *Pbk*$^{+/+}$ and *Pbk*$^{-/-}$ mouse preB cells.

a. Fragment length distribution in merged *Pbk*$^{+/+}$ and *Pbk*$^{-/-}$ mitotic chromosome ATAC-seq libraries after alignment (*n*=4+4, log10 scale, 5 bp rolling average).

b. Principal component analysis of ATAC-seq libraries based on variance-stabilising transformed read counts in consensus MACS2 peaks.

c. Representative loci showing nucleosome-free (<100bp) ATAC-seq signal from *Pbk*$^{+/+}$ (blue) and *Pbk*$^{-/-}$ (orange) mitotic chromosomes (normalised merged signal, *n*=4+4). Published asynchronous CTCF peaks[61] and consensus ATAC-seq peaks shown below (none significantly altered in these regions).

d. Individual replicate tracks of nucleosome-free (≤100bp) ATAC-seq signal at loci shown in Fig. 5d. Published CTCF peaks[61] and consensus ATAC-seq peaks shown underneath (significantly altered peaks in red, $p_{adj}<0.1$); asterisks mark loci for CTCF ChIP-qPCR (Supplementary Fig. 5i).

e. GO term enrichment (biological process) amongst genes with increased (upper; top ten significant terms) or decreased (bottom) TSS accessibility.

f. Top ten enriched motifs in peaks with decreased accessibility in *Pbk*$^{-/-}$ mitotic chromosomes; only ZBTB11 is a C2H2-ZF protein.

g. ATAC-seq footprint profiles for three motifs showing the largest changes in score between *Pbk*$^{+/+}$ and *Pbk*$^{-/-}$ mitotic chromosomes (one representative CTCF motif is shown). Bias-corrected TOBIAS score plotted ±60 bp from motif centre (grey shading), smoothed using 5 bp rolling average.

h. FACS gating to purify mitotic cells for ChIP. Demecolcine-arrested, fixed cells were stained with PI and anti-H3S10p Alexa Fluor 488. Mitotic cells were gated by 4N DNA (PI) and high H3S10p. Mean mitotic percentages (±SD) shown for *Pbk*$^{+/+}$ (*n*=6) and *Pbk*$^{-/-}$ (*n*=5) sorts (we note reduced G2/M percentage after formaldehyde versus ethanol fixation (compare Supplementary Fig. 4b)).

i. CTCF ChIP-qPCR enrichment (% of input) in asynchronous and purified mitotic *Pbk*$^{+/+}$ and *Pbk*$^{-/-}$ mouse preB cells, at known CTCF peaks and negative control sites (*Ccdc110* and *Tmcc2* loci are marked in Fig. 5d and Supplementary Fig. 5d). *N*=3 (mitotic cells pooled from multiple sorts where necessary, one WT asynchronous replicate excluded, details in Methods); geometric mean with geometric SD; two-sided *t*-tests on adjusted delta-Ct values (assuming homogeneity of variance) with Holm-Šídák's correction (****$p_{adj}<0.0001$, ***$p_{adj}<0.001$, **$p_{adj}<0.01$).

Source data for Supplementary Figs. 5h-i are provided as a Source Data file.

Supplementary Figure 6

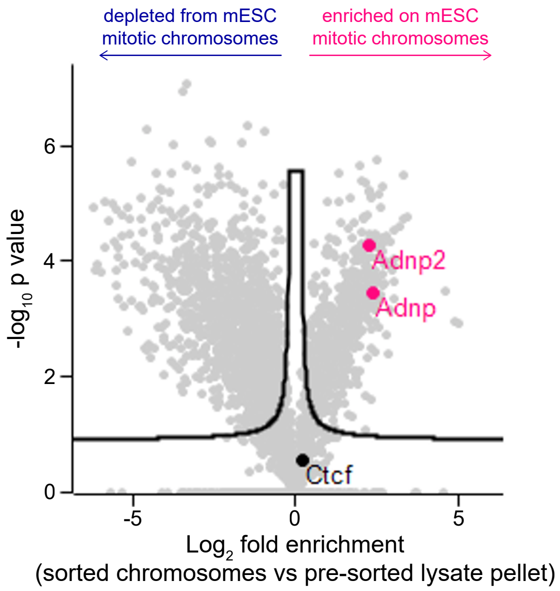

Supplementary Figure 6 | Mitotic chromosome enrichment of CTCF and ADNP/ADNP2 in mESCs.

LC-MS/MS proteomic analysis of mESC mitotic chromosomes taken from[10], highlighting CTCF (not significantly enriched) and ADNP/ADNP2 (significantly enriched); modified two-tailed *t*-test with permutation-based FDR<0.01, S0=0.1, *n*=3.

Supplementary Tables

Table 1 | Oligonucleotides for engineering and validating KI and KO cells.

Single guide RNA (sgRNA) sequences for CRISPR/Cas9 editing were selected using the UCSC genome browser (https://genome.ucsc.edu/)[101,102]. Primers for *Ikzf1-mNeonGreen* donor plasmid construction were designed using Primer3Plus (https://www.primer3plus.com/, v3.1.0)[128] and the NEBuilder Assembly Tool (https://nebuilder.neb.com, v2.3.0). Primers for PCR validation were designed using Primer3Plus (https://www.primer3plus.com/, v3.1.0 and v3.2.0)[128].

Guide RNAs

Purpose	Oligo sequences	Notes
sgRNA for *Ikzf1-mNeonGreen* KI (1)	caccgCTGGCTGGGTTTAGCTCAGG<br>aaacCCTGAGCTAAACCCAGCCAGc	Guides target 3' end of *Ikzf1* or third coding exon of *Pbk*. Guide sequence in caps; upstream G added if not present in the guide (increases efficiency of U6 transcription) along with overhangs for golden gate assembly (lower case).
sgRNA for *Ikzf1-mNeonGreen* KI (2)	caccgCACCTGAGCTAAACCCAGCC<br>aaacGGCTGGGTTTAGCTCAGGTGc	
sgRNA for PBK KO	caccGTATATTTTAGATCTCCAAG<br>aaacCTTGGAGATCTAAAATATAC	

***Ikzf1-mNeonGreen* donor plasmid construction**

Component	Oligo sequences[a]	Template source
Backbone incl. EBFP2 gene (3519 bp)	atcctggttcctgttcttccccgagTAAGCTTGGGCCGCTCGAG<br>gggataaggtgccatctttcccaggTTCCTGCCCGACCTTGGTAC	Plasmid gifted by Stefan Stricker[129]
Ikzf1 left homology arm (820 bp to final codon)	CCTGGGAAAGATGGCACC<br>GCTCAGGTGGTAACGATG	Genomic DNA
Spacer (underlined) plus *mNeonGreen* (720 bp)	gggggagcatcgttaccacctgagc<u>ggaggtggttct</u>GTGAGCAAGGGCGAGGAG<br>gtgcttcagtggggcctggctgggtTTACTTGTACAGCTCGTCCATG	3xnls-mNeonGreen (Addgene #98875), gift from Dorus Gadella[130]
Ikzf1 right homology arm (880 bp downstream)	ACCCAGCCAGGCCCCACTG<br>CTCGGGGAAGAACAGGAACCAGG	Genomic DNA

Verification of CRISPR/Cas9 engineered cells

Purpose	Oligo sequences	Expected sizes
Ikzf1 WT versus *mNeonGreen* KI (1)	GGCTTTCGGGATCCCTTTGA<br>TCCACTCCCAACATTGTCCG	WT=197 bp<br>KI=914 bp (for sanger sequencing)
Ikzf1 WT versus *mNeonGreen* KI (2)	CCTGGGAAAGATGGCACC<br>CTCGGGGAAGAACAGGAACCAGG	WT=1703 bp (for sanger sequencing)<br>KI=2420 bp
Pbk KO	TGGAGCAAAATTTGAGTGTTGG<br>ACCACATACTGCCACAAAGT	WT=248 bp

[a]Upper case letters anneal to the template, lower case letters provide overlaps for assembly.

Table 2 | Antibodies used in this study.

Target	Antibody details	Use	Dilution
Ikaros	Rabbit antisera to C-terminal Ikaros[27], gifted by Stephen Smale	Western blot, primary<br>IF, primary	1:1,000-5,000<br>1:200
PBK	Abcam rabbit monoclonal anti-PBK [EPR21983], ab236872	Western blot, primary	1:1,000
Phosphorylated linker peptide<br>KRSH(Tp)GER	Covalab custom 'anti-phospho-linker' antibody. Rabbit polyclonal antibody was raised by initial and booster injections of phospho-peptides (C-MVHKRSH(Tp)GERPFQ-coNH2; C-KRSH(Tp)GER-coNH2). Resulting serum was depleted with a non-phospho-peptide (MVHKRSHTGERPFQ-coNH2), followed by purification with the short phospho-peptide. Affinity and specificity were verified by ELISA (Covalab).	Western blot, primary<br>IP	1:1,000<br>4 µg / mg protein
Histone H3S10p	Abcam rabbit polyclonal anti-Histone H3 (phospho S10), ab5176	Western blot, primary	1:5,000
	Alexa Fluor 488 mouse monoclonal anti-Histone H3 (phospho S10) [mAbcam 14955], ab197502	FACS, conjugated primary	1:1,000
Histone H3	Abcam rabbit polyclonal anti-Histone H3, ab1791	Western blot, primary	1:8,000-10,000
GAPDH	Abcam mouse monoclonal anti-GAPDH [6C5], ab8245	Western blot, primary	1:2,000
CTCF	Cell Signaling Technology rabbit polyclonal anti-CTCF, #2899	Western blot<br>IF, primary	1:1,000<br>1:100
	Abcam rabbit recombinant monoclonal CTCF antibody [EPR7314(B)], ab128873	ChIP	3 µg / 4 x 10^6 asynch. cells<br>1.7 µg / 7 x 10^5 mitotic cells
SP1	Invitrogen rabbit monoclonal anti-SP1 [ARC0128], MA5-35331	Western blot<br>IF, primary	1:500<br>1:50
	Abcam rabbit polyclonal anti-SP1, ab227383	IF, primary (additional validation)	1:100
YY1	Abcam rabbit monoclonal anti-YY1 [EPR4652], ab109237	Western blot<br>IF, primary	1:2,000<br>1:50
Rabbit IgG (H+L)	Invitrogen goat anti-Rabbit IgG (H+L) Alexa Fluor 680, A-21109<br>Invitrogen goat anti-Rabbit IgG (H+L) Alexa Fluor 633, A-21070	Western blot, secondary<br>IF, secondary	1:10,000<br>1:500
Mouse IgG (H+L)	Invitrogen goat anti-Mouse IgG (H+L) Alexa Fluor 680, A-21057	Western blot, secondary	1:10,000

Table 3 | Kinase and phosphatase inhibitors used in this study.

Inhibitor	Source(s)	Main target(s)	Concentration
K252a	Abcam, ab120419<br>Enzo Life Sciences (BioVision), 2013-1000	Broad, non-selective protein kinase inhibitor	1 µM
OTS514 hydrochloride	Stratech (Selleck Chemicals), S7652-SEL	PBK (also known as TOPK)	10 µM
VX-680 (also known as MK-0457 or Tozasertib)	Stratech (ApexBio), A4111-APE	Aurora kinases (and wide array of other kinases)	10 µM
Alsterpaullone (Alp)	Stratech (ApexBio), B7855-APE	Cyclin dependent kinases (CDKs), GSK3β	10 µM
CX-4945 (also known as Silmitasertib)	Stratech (ApexBio), A8330-APE	Casein kinase 2 (CK2)	10 µM
KN-62	Stratech (Selleck Chemicals), S7422-SEL	CaMKII (also CaMKI, P2RX7, CaMKIV)	10 µM
Chelerythrine Chloride	Stratech (ApexBio), A3306-APE	Protein kinase C (PKC)	10 µM
Akt Inhibitor VIII	Merck (Calbiochem), 124017	Akt1/2	10 µM
Saracatinib (also known as AZD0530)	Stratech (ApexBio), A2133-APE	Src family kinases (also Abl and EGFR)	10 µM
Hesperadin	Merck (Calbiochem), 375680	Aurora kinase B	10 µM
OTS964	Stratech (Selleck Chemicals), S7648-SEL	PBK (also known as TOPK), CDK11B	10 µM
Okadaic acid (OA)	Merck, O9381<br>Cell Signaling, 5934S	Protein phosphatase PP2A (also PP1 at lower potency)	1 µM
Calyculin A	Merck (Calbiochem), 5082260001<br>Abcam, ab141784	Protein phosphatases PP1 and PP2A	0.1 µM

Table 4 | Primers for ATAC-seq library amplification.

Primer sequences were taken from[131] and ordered with HPLC purification (Sigma-Aldrich).

Primer	**Sequence** (unique index underlined)	Library
Ad1	AATGATACGGCGACCACCGAGATCTACACTCGTCGGCAGCGTCAGATGTG	All
Ad2.1	CAAGCAGAAGACGGCATACGAGATTCGCCTTAGTCTCGTGGGCTCGGAGATGT	*Pbk*$^{+/+}$ rep1
Ad2.2	CAAGCAGAAGACGGCATACGAGATCTAGTACGGTCTCGTGGGCTCGGAGATGT	*Pbk*$^{-/-}$ rep1
Ad2.3	CAAGCAGAAGACGGCATACGAGATTTCTGCCTGTCTCGTGGGCTCGGAGATGT	*Pbk*$^{+/+}$ rep2
Ad2.4	CAAGCAGAAGACGGCATACGAGATGCTCAGGAGTCTCGTGGGCTCGGAGATGT	*Pbk*$^{-/-}$ rep2
Ad2.5	CAAGCAGAAGACGGCATACGAGATAGGAGTCCGTCTCGTGGGCTCGGAGATGT	*Pbk*$^{+/+}$ rep3
Ad2.6	CAAGCAGAAGACGGCATACGAGATCATGCCTAGTCTCGTGGGCTCGGAGATGT	*Pbk*$^{-/-}$ rep3
Ad2.7	CAAGCAGAAGACGGCATACGAGATGTAGAGAGGTCTCGTGGGCTCGGAGATGT	*Pbk*$^{+/+}$ rep4
Ad2.8	CAAGCAGAAGACGGCATACGAGATCCTCTCTGGTCTCGTGGGCTCGGAGATGT	*Pbk*$^{-/-}$ rep4

Table 5 | Primers for CTCF ChIP-qPCR.

Primers were designed using Primer3web (https://primer3.ut.ee, v4.1.0)[128].

Target region	CTCF peak?	Primers	
Ccdc110	Yes	F: GTGTGCGTTATCTTCCCGAC	R: CTGCAAATAGGGGTGAAGGC
Cdc20b	Yes	F: AGAAGCATGCAAAGACGTGG	R: TTGGGTTAGAGCTTTAAGGGG
Zfp444/Galp	Yes	F: ACAGAGAGAGACAGCAGCTC	R: TCCACATTTCTTCCCAGTGTTC
Krr1	Yes	F: CTCACCTTGCAATCCACACA	R: CTACTGGTTGCACGCGTTAC
Tmcc2 3'	Yes	F: AGAGAGGTTTTGGAGCACGG	R: GAAGGGAAATGGAGAAGCGC
Tmcc2 promoter 1 (p1)	Yes	F: AAAGATGGCCAACTCACAGC	R: GCTCCCTCTGCTGACCAATA
Tmcc2 promoter 2 (p2)	No	F: GGGGAGCCTTAGGATAGTTCA	R: CCAGTGACTGCCTAAGGACC
Cdx2	No	F: ACCACCTTCTGCCTGAGAATGTAC	R: CCTCCAATCACAGGTTCAAAGACT
Chr19 gene desert	No	F: TGCATGAGCAGAGGACTAGG	R: AGAAGTGCAAGCTCAGAACCTT

Supplementary References

128. Untergasser, A. *et al.* Primer3—new capabilities and interfaces. *Nucleic Acids Res.* **40**, e115 (2012).

129. Breunig, C. T. *et al.* One step generation of customizable gRNA vectors for multiplex CRISPR approaches through string assembly gRNA cloning (STAgR). *PLoS One* **13**, e0196015 (2018).

130. Chertkova, A. O. *et al.* Robust and Bright Genetically Encoded Fluorescent Markers for Highlighting Structures and Compartments in Mammalian Cells. *bioRxiv* 160374 (2020) doi:10.1101/160374.

131. Buenrostro, J. D., Giresi, P. G., Zaba, L. C., Chang, H. Y. & Greenleaf, W. J. Transposition of native chromatin for fast and sensitive epigenomic profiling of open chromatin, DNA-binding proteins and nucleosome position. *Nat. Methods* **10**, 1213–8 (2013).

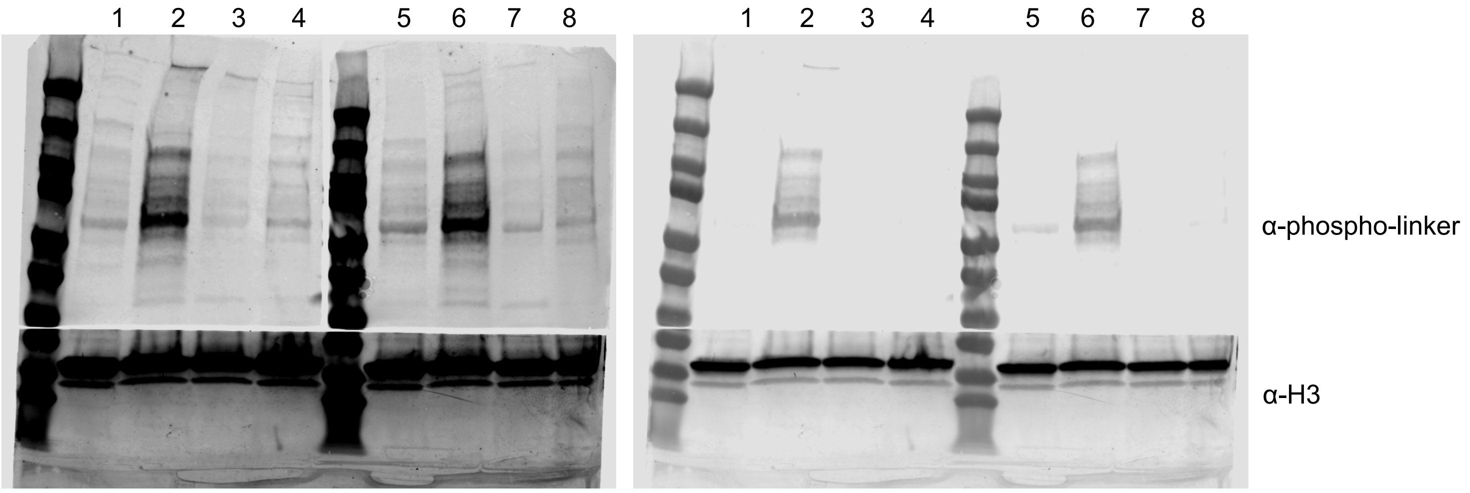

Uncropped blots for Supplementary Fig. 4c.

Left and right images show the same blot image with brightness/contrast adjusted for display of phospho-linker or H3 signal respectively. Lanes 1-3 were used to prepare Supplementary Fig. 4c, where lane 1=WT asynchronous; 2=WT demecolcine-arrested; 3=WT demecolcine-arrested + OTS514 (10 µM). Lane 4 shows asynchronous Ikaros-mNeonGreen KI preB cells (clone2.1); lanes 5-8 are the same samples, but probed with a less purified version of the phospho-linker antibody.

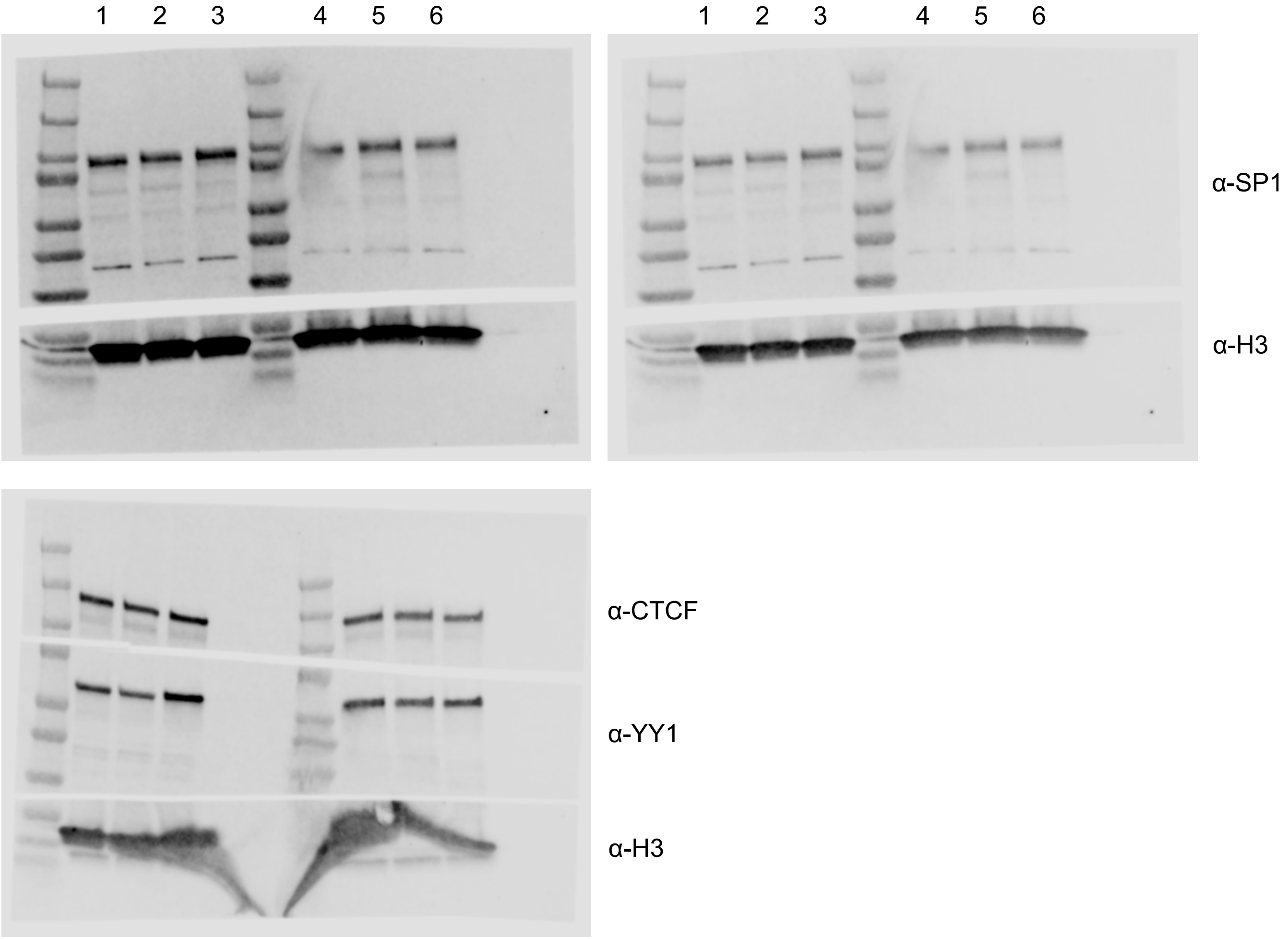

Uncropped blots for Supplementary Fig. 4f.

Top left and right images show the same blot image with brightness/contrast adjusted for display of SP1 or H3 signal respectively. Top and bottom gels were loaded with the same samples/volumes and run at same time. Lanes 1-2 were used to prepare Supplementary Fig. 4f, where lane 1=*Pbk*$^{+/+}$ and lane 2=*Pbk*$^{-/-}$. Lane 3 is from a second *Pbk*$^{-/-}$ clone. Lanes 4-6 are independently collected lysates from the same cell lines as lanes 1-3.
