# Supplementary material for: PBK/TOPK mediates Ikaros, Aiolos and CTCF displacement from mitotic chromosomes and alters chromatin accessibility at selected C2H2-zinc finger protein binding sites
Source: Nat Commun. 2025 Sep 23;16:8348. doi: 10.1038/s41467-025-63740-4 (PMC12457614; doi:10.1038/s41467-025-63740-4)
Supplement: Supplementary file 2 — Description of Additional Supplementary Files [file 41467_2025_63740_MOESM2_ESM.pdf]

Description of Additional Supplementary Files

Supplementary Data 1

Corresponding to Fig. 3e and Supplementary Figs. 3e-f.

Protein group files for LC-MS/MS proteomics on flow-sorted mitotic chromosomes and pre-sorted mitotic lysate pellets (LP) for *Pbk*$^{+/+}$ (WT, Tab 1) and *Pbk*$^{–/–}$ (KO, Tab 2) mouse preB cells.

Supplementary Data 2

Corresponding to Fig. 4c and Supplementary Fig. 4a.

Protein group files for LC-MS/MS proteomics analysis of *Pbk*$^{+/+}$ and *Pbk*$^{–/–}$ mouse preB mitotic lysate inputs

(Tab 1) and following immunoprecipitation with anti-phospho-linker antibody (Tab 2).

Supplementary Movies 1-4 | Live-cell imaging of Ikaros-mNeonGreen through mitosis.

Corresponding to Figs. 1b-c and Supplementary Fig. 1c.

Time-lapse live-cell imaging of Ikaros-mNeonGreen (middle panel, green) localisation through mitosis at 3 min intervals in KI mouse preB cell clones pre-incubated with SiR-DNA (left panel, magenta). Movie 1=clone 1.1; Movie 2=clone 1.2; Movie 3=clone 2.1; Movie 4=clone 2.2; scale bars=5 µm.
