# Supplementary material for: PBK/TOPK mediates Ikaros, Aiolos and CTCF displacement from mitotic chromosomes and alters chromatin accessibility at selected C2H2-zinc finger protein binding sites
Source: Nat Commun. 2025 Sep 23;16:8348. doi: 10.1038/s41467-025-63740-4 (PMC12457614; doi:10.1038/s41467-025-63740-4)
Supplement: Supplementary file 9 — Reporting Summary [file 41467_2025_63740_MOESM9_ESM.pdf]

nature portfolio

Corresponding author(s): Amanda Fisher, Andrew Dimond

Last updated by author(s): Jul 24, 2025

Reporting Summary

Nature Portfolio wishes to improve the reproducibility of the work that we publish. This form provides structure for consistency and transparency in reporting. For further information on Nature Portfolio policies, see our Editorial Policies and the Editorial Policy Checklist.

Statistics

For all statistical analyses, confirm that the following items are present in the figure legend, table legend, main text, or Methods section.

n/a	Confirmed	
☐	☒	The exact sample size (*n*) for each experimental group/condition, given as a discrete number and unit of measurement
☐	☒	A statement on whether measurements were taken from distinct samples or whether the same sample was measured repeatedly
☐	☒	The statistical test(s) used AND whether they are one- or two-sided<br>*Only common tests should be described solely by name; describe more complex techniques in the Methods section.*
☐	☒	A description of all covariates tested
☐	☒	A description of any assumptions or corrections, such as tests of normality and adjustment for multiple comparisons
☐	☒	A full description of the statistical parameters including central tendency (e.g. means) or other basic estimates (e.g. regression coefficient) AND variation (e.g. standard deviation) or associated estimates of uncertainty (e.g. confidence intervals)
☐	☒	For null hypothesis testing, the test statistic (e.g. *F*, *t*, *r*) with confidence intervals, effect sizes, degrees of freedom and *P* value noted<br>*Give P values as exact values whenever suitable.*
☒	☐	For Bayesian analysis, information on the choice of priors and Markov chain Monte Carlo settings
☒	☐	For hierarchical and complex designs, identification of the appropriate level for tests and full reporting of outcomes
☐	☒	Estimates of effect sizes (e.g. Cohen's *d*, Pearson's *r*), indicating how they were calculated

Our web collection on statistics for biologists contains articles on many of the points above.

Software and code

Policy information about availability of computer code

Data collection

BD FACS Sortware (v1.2.0.142) and BD FACSDiva Software (v9.1 and v9.4) were use to collect flow cytometry data.
Micro-Manager (v2.0, IX70 microscope), cellSens Dimension (v2.3, IX83 microscope), LAS-AF (v2.7.3.9723, Leica SP5 II confocal microscope) were used to collect imaging data.
Image Studio software (v5.2.5 or v5.5) was used for fluorescent western blot imaging.
ATAC-seq sequencing data were collected on an Illumina NextSeq 2000 with NextSeq 1000/2000 Control Software (v1.4.1.39716).
Quantitative real-time PCR data was collected using Bio-Rad CFX Manager software (v3.1).

Data analysis

FlowJo software (v10.8.1) was used to analyse PI staining data and create plots.
Image deconvolution was performed using Huygens Professional software (Scientific Volume Imaging, v19.10).
Volume analysis of z-stack images was performed using the surfaces and cells packages in Imaris (Bitplane, v10.0.0).
Chromosome spread images were segmented using Cellpose (v2.0). Chromosome paint classification and area measurements were subsequently performed with QuPath (v0.4.3).
Fiji (1.54e) was used for chromosome size analyses and for preparing all cell/chromosome imaging figures.
Image Studio Lite (v5.2) was used to process western blot images.
Raw proteomics data were analyzed using the MaxQuant software platform (v1.6.10.43, Label-Free Quantification algorithm) and Perseus software (v1.6.15.0) was used for downstream statistical analysis and visualisation. Correlation values were visualised as a heatmap using the R package corrplot (v0.92). Normalised values from the IP experiment were visualised as a heatmap using Microsoft Excel 365.
GO term overrepresentation analysis was performed using PANTHER (v18.0).
For ATAC-seq data generated in this study, primary data analysis was performed with RTA (v3.9.25), secondary analysis with DRAGEN Generate FastQ (v3.7.4), and reads were demultiplexed with bcl2fastq2 (v2.20). Reads were trimmed with fastp (v0.23.3), aligned with bwa-mem (v0.7.17) and filtered using samtools (v1.17). PCR duplicates were marked using sambamba (v1.0.1) and replicates were merged using

samtools (v1.17). Fragment size distributions were calculated using deeptools bamPEFragmentSize (v3.5.1). Peaks were called using MACS2 callpeak (v2.2.8). The GenomicRanges R package (v1.46.1) was used to shift reads, merge consensus peaks and generate overlapping counts. Differential accessibility analysis was performed with DESeq2 (v1.34.0). Motif enrichment analysis was performed with AME (MEME suite v5.5.4) using JASPAR 2024 CORE vertebrates non-redundant motifs. GO term enrichment analysis was carried out using clusterProfiler (v4.2.2). Transcription factor footprinting analysis was carried out using TOBIAS (v0.16.0) with JASPAR 2024 CORE vertebrates non-redundant motifs.
Published CTCF ChIP-seq data were download from SRA (fasterq-dump, sratools v3.0.3), aligned using bowtie2 (v2.5.1), filtered using samtools (v1.16.1), and PCR duplicates were marked using sambamba (v1.0.1). Peaks were called using MACS2 (v2.2.8). CTCF motif locations were identified using the motifmatchr R package (v1.16.0) and the MA0139.2 motif from JASPAR2024.
Published ATAC-seq data were download from SRA (fasterq-dump, sratools v3.0.3), trimmed using fastp (v0.23.3), aligned using bowtie2 (v2.5.1), filtered using samtools (v1.16.1), and PCR duplicates were marked using sambamba (v1.0.1).
For sequencing data, heatmaps were plotted using deeptools (v3.5.1), coverage tracks were created with deeptools bamCoverage (v3.5.1), and tracks at example loci were visualised using the IGV genome browser (v2.9.2). Nucleosome positioning plots were produced using deeptools computeMatrix and plotted using ggplot2.
Quantitative real-time PCR data was analysed using Bio-Rad CFX Manager software (v3.1) and Microsoft Excel 365.
GraphPad Prism (v10.1.1) was used for preparing graphs and performing statistical analyses.
The UCSC genome browser (https://genome.ucsc.edu/) was used to select gRNA sequences.
Primers were designed using Primer3Plus (https://www.primer3plus.com/, v3.1.0 and v3.2.0), Primer3web (https://primer3.ut.ee, v4.1.0) and the NEBuilder Assembly Tool (https://nebuilder.neb.com, v2.3.0).

For manuscripts utilizing custom algorithms or software that are central to the research but not yet described in published literature, software must be made available to editors and reviewers. We strongly encourage code deposition in a community repository (e.g. GitHub). See the Nature Portfolio guidelines for submitting code & software for further information.

Data

Policy information about availability of data

All manuscripts must include a data availability statement. This statement should provide the following information, where applicable:

- Accession codes, unique identifiers, or web links for publicly available datasets
- A description of any restrictions on data availability
- For clinical datasets or third party data, please ensure that the statement adheres to our policy

Mass spectrometry proteomics data have been deposited to the ProteomeXchange Consortium via the PRIDE partner repository with the dataset identifiers PXD057894 [https://www.ebi.ac.uk/pride/archive/projects/PXD057894] and PXD057898 [https://www.ebi.ac.uk/pride/archive/projects/PXD057898]; processed data are provided in Supplementary Data 1 and 2. ATAC-seq data have been deposited to GEO with accession number GSE266554 [https://www.ncbi.nlm.nih.gov/geo/query/acc.cgi?acc=GSE266554]. Original image stacks have been uploaded to Figshare with DOI 10.6084/m9.figshare.29627411 [https://doi.org/10.6084/m9.figshare.29627411]. The UCSC genome browser guide RNA track BED file is available from https://hgdownload.soe.ucsc.edu/gbdb/mm10/crisprAll/crispr.bb. Published CTCF ChIP-seq data (Rep1: SRR6512735, SRR6512736; Rep2: SRR6512737, SRR6512738; Input: SRR6512739, SRR6512740) and ATAC-seq data (SRR6512723, SRR6512724, SRR6512725, SRR6512726) were downloaded from the NCBI SRA (SRP131401, corresponding to GEO accession GSE109671 [https://www.ncbi.nlm.nih.gov/geo/query/acc.cgi?acc=GSE109671]). JASPAR 2024 CORE vertebrate non-redundant data are available from https://jaspar.elixir.no/downloads/. GO annotations for use in Perseus were downloaded from http://annotations.perseus-framework.org (mainAnnot.mus_musculus.txt). The GO Ontology database used within PANTHER is identifiable with DOI: 10.5281/zenodo.8436609 [https://doi.org/10.5281/zenodo.8436609]. Data from UniProt (https://www.uniprot.org/) and the MGI Gene Ontology Browser (https://www.informatics.jax.org/function.shtml) were used for additional manual annotations where indicated. Source data for Figs. 1b, 1d, 2a, 2c, 2e-f, 3c-d, 4b and Supplementary Figs. 4b-c, 4f-g and 5h-i are provided with this paper. All other data supporting the key findings of this study are available within the article and its Supplementary files. Any further details and unique biological materials are available from the authors upon reasonable request.

Research involving human participants, their data, or biological material

Policy information about studies with human participants or human data. See also policy information about sex, gender (identity/presentation), and sexual orientation and race, ethnicity and racism.

Reporting on sex and gender	*Use the terms sex (biological attribute) and gender (shaped by social and cultural circumstances) carefully in order to avoid confusing both terms. Indicate if findings apply to only one sex or gender; describe whether sex and gender were considered in study design; whether sex and/or gender was determined based on self-reporting or assigned and methods used. Provide in the source data disaggregated sex and gender data, where this information has been collected, and if consent has been obtained for sharing of individual-level data; provide overall numbers in this Reporting Summary. Please state if this information has not been collected. Report sex- and gender-based analyses where performed, justify reasons for lack of sex- and gender-based analysis.*
Reporting on race, ethnicity, or other socially relevant groupings	*Please specify the socially constructed or socially relevant categorization variable(s) used in your manuscript and explain why they were used. Please note that such variables should not be used as proxies for other socially constructed/relevant variables (for example, race or ethnicity should not be used as a proxy for socioeconomic status). Provide clear definitions of the relevant terms used, how they were provided (by the participants/respondents, the researchers, or third parties), and the method(s) used to classify people into the different categories (e.g. self-report, census or administrative data, social media data, etc.) Please provide details about how you controlled for confounding variables in your analyses.*
Population characteristics	*Describe the covariate-relevant population characteristics of the human research participants (e.g. age, genotypic information, past and current diagnosis and treatment categories). If you filled out the behavioural & social sciences study design questions and have nothing to add here, write "See above."*
Recruitment	*Describe how participants were recruited. Outline any potential self-selection bias or other biases that may be present and*

Recruitment	*how these are likely to impact results.*
Ethics oversight	*Identify the organization(s) that approved the study protocol.*

Note that full information on the approval of the study protocol must also be provided in the manuscript.

Field-specific reporting

Please select the one below that is the best fit for your research. If you are not sure, read the appropriate sections before making your selection.

☒ Life sciences ☐ Behavioural & social sciences ☐ Ecological, evolutionary & environmental sciences

For a reference copy of the document with all sections, see nature.com/documents/nr-reporting-summary-flat.pdf

Life sciences study design

All studies must disclose on these points even when the disclosure is negative.

Sample size	We used minimum n=3 for proteomics experiments to allow t-test comparisons in Perseus; this was sufficient in previously published work (Djeghloul et al., 2020; Djeghloul et al., 2023) to identify multiple differentially enriched factors on mitotic chromosomes.<br>ATAC-seq was performed with n=4, with a total of ~300 million mapped reads per condition - this provides sufficient read-depth for differential accessibility analyysis and footprinting analysis (community standards recommend >50 million and >200 million respectively).<br>For experiments involving imaging cells or chromosomes, analysis is from at least two independent experiments to ensure reproducibility; most experiments are from three or more independent experiments. The number of cells or chromosomes is specified for each graph and was determined in part by practical constraints on image acquisition. Numbers of cells and chromosomes imaged is comparable to previously published work (Djeghloul et al., 2020; Djeghloul et al., 2023) where statistical differences in chromosome sizes could be detected.<br>Western blots were performed for a minimum of two independent biological replicates to ensure reproducibility; this is a common community standard.<br>ChIP-qPCR experiments were performed in biological triplicate to ensure reproducibility and allow statistical comparisons; this is a common community standard.
Data exclusions	Live or fixed cell imaging: Some replicates showed unexplained widespread cell death, or cells appeared to have been damaged during sample preparation. These replicates or cells were excluded.<br>Chromosome size measurements: Clumped, overlapping or obviously damaged chromosomes were excluded; centromeres which were not clearly defined were excluded<br>Proteomics: Two pre-sorted lysate pellet samples (Pbk+/+ replicate 1 and Pbk–/– replicate 4) exhibited outlier retention time distributions in the mass spec data and were excluded from the analysis.<br>ATAC-seq: Peaks and data on chromosomes 6 and 14 were excluded from downstream analysis because by visual inspection these chromosomes have globally reduced ATAC-seq coverage in Pbk–/–.<br>Previously published CTCF ChIP-seq data: Very few peaks were called for Rep2 and so this replicate was excluded from downstream analysis.<br>One WT asynchronous ChIP-qPCR sample was excluded on the basis of outlier values at negative control regions in the qPCR.
Replication	All imaging experiments were performed using a minimum of two independent biological replicates (most from three or more), with images of multiple cells acquired for each to ensure that images are representative. Details of the number of cells imaged and any variability between cells is reported in the figure legends.<br>Live-cell imaging of Ikaros localisation was confirmed in four independent mNeonGreen knock-in clones. The impact of OTS514 treatment on Ikaros localisation was replicated in a second mNeonGreen knock-in clone. Key results from Pbk–/– cells were replicated in a second knock-out clone, including retention of Ikaros & CTCF, and loss of phospho-linker detection in mitosis.<br>Western blots were performed for a minimum of two independent biological replicates.<br>Proteomics analyses were performed using minimum n=3 three independent chromosome sorts)<br>ATAC-seq was performed using n=4 (four independent chromosome sorts)<br>ChIP qPCR measurements were performed in technical duplicate for three independent biological replicates.<br>All replicate attempts were successful, with the exception of the exclusions noted above.
Randomization	Randomization was not relevant as there was no assignment of samples to different experimental groups (all experiments conducted on cell lines).
Blinding	Blinding was not relevant since there was no assignment of samples to different experimental groups (all experiments conducted on cell lines).

Reporting for specific materials, systems and methods

We require information from authors about some types of materials, experimental systems and methods used in many studies. Here, indicate whether each material, system or method listed is relevant to your study. If you are not sure if a list item applies to your research, read the appropriate section before selecting a response.

Materials & experimental systems

n/a	Involved in the study
☐	☒ Antibodies
☐	☒ Eukaryotic cell lines
☒	☐ Palaeontology and archaeology
☒	☐ Animals and other organisms
☒	☐ Clinical data
☒	☐ Dual use research of concern
☒	☐ Plants

Methods

n/a	Involved in the study
☒	☐ ChIP-seq
☐	☒ Flow cytometry
☒	☐ MRI-based neuroimaging

Antibodies

Antibodies used

Primary antibodies:
Rabbit antisera to C-terminal Ikaros (Hahm et al, Mol Cell Biol, 1994), gifted by Stephen Smale.
Anti-PBK/SPK monoclonal antibody [EPR21983], Abcam ab236872, lot GR3243503
Anti-phospho linker (target peptide = KRSH(Tp)GER), custom antibody generated by Covalab (details provided in manuscript)
Anti-Histone H3 (phospho S10) polyclonal antibody, Abcam ab5176
Anti-Histone H3 (phospho S10) monoclonal antibody conjugated to Alexa Fluor 488 [mAbcam 14955], Abcam ab197502, lot 1027931-8
Anti-Histone H3 polyclonal antibody, Abcam ab1791
Anti-GAPDH monoclonal antibody [6C5], Abcam ab8245
Anti-CTCF polyclonal antibody, Cell Signaling Technology #2899
Anti-CTCF rabbit recombinant monoclonal antibody [EPR7314(B)], ab128873, lot 1085805-10
Anti-SP1 recombinant monoclonal antibody [ARC0128], Invitrogen MA5-35331
Anti-SP1 polyclonal antibody, Abcam ab227383, lot GR3346147
Anti-YY1 recombinant monoclonal antibody [EPR4652], Abcam ab109237, lot 1001564-7

Secondary antibodies:
Goat anti-Rabbit IgG (H+L) Highly Cross-Adsorbed Secondary Antibody, Alexa Fluor™ 680, Thermo Fisher Scientific (Invitrogen) A-21109
Goat anti-Rabbit IgG (H+L) Highly Cross-Adsorbed Secondary Antibody, Alexa Fluor™ 633, Thermo Fisher Scientific (Invitrogen) A-21071
Goat anti-Mouse IgG (H+L) Cross-Adsorbed Secondary Antibody, Alexa Fluor™ 680, Thermo Fisher Scientific (Invitrogen) A-21057

Validation

Antisera to C-terminal Ikaros:
-described in Hahm et al, Mol Cell Biol, 1994; suitable for WB, reacts with mouse Ikaros.
-shown to be specific for Ikaros over Helios (Hahm et al, Genes Dev, 1998).
-shown to be suitable for IF in mouse cells in Brown et al, Cell, 1997; Cobb et al, Genes Dev, 2000.
-specificity supported by detection of over-expressed Ikaros (Cobb et al, Genes Dev, 2000; Thompson et al, Immunity, 2007).

Anti-PBK [EPR21983]:
-supplier website: tested for WB in mouse; validated on PBK KO cells.

Anti-phospho linker (Covalab):
-affinity and specificity to phospho linker (versus control peptide) was validated by ELISA (Covalab).
-WB and IP+mass spec experiments in this study (Figs. 4b-c) using WT and PBK KO cells confirm detection of phosphorylated PBK targets. Comparison of protein levels in input samples confirms specific detection of phosphorylated (but not unphosphorylated) forms (Supplementary Fig 4a).

Anti-Histone H3 (phospho S10) (ab5176):
-supplier website: suitable for WB, predicted to react with mouse. Specificity - this antibody is specific for phosho S10 of histone H3. Should not recognise the non-modified histone - no blocking is seen with the non-phospho peptide.
-previously validated for use in mouse cells by IF comparing interphase and metaphase-arrested cells (Djeghloul et al., Nat Struct Mol Biol, 2023).
-WB in this study shows greatly increased signal in mitotically-arrested cells as expected (Fig. 4c).

Anti-Histone H3 (phospho S10) - Alexa Fluor 488 [mAbcam 14955]:
-supplier website: suitable/tested for Flow Cytometry (Intra); predicted to react with mouse. The non-conjugated version has been tested to react with mouse in ICC/IF. Specificity - this clone binds to tri-methyl K9/phospho S10 dimodified peptides and to a phospho S10 peptide, but not to a tri-methyl K9 peptide or to unmodified Histone H3 peptide.
-flow cytometry analysis performed for this study showed the expected staining pattern in conjunction with PI stain (Supplementary Fig. 5h, only a sub-population of 4N cells show distinct staining); this positively stained population increases after mitotic arrest as expected.

Anti-Histone H3 (ab1791):
-supplier website: tested for WB in mouse; cited in 4737 publications.

Anti-GAPDH [6C5]:
-supplier website: used in WB, suitable for mouse samples. "Antibody clone 6C5 is the most widely used clone for GAPDH on the market and is cited in >15630 publications". "This GAPDH antibody can be used as a loading control antibody."

Anti-CTCF (#2899):
-supplier website: suitable for WB and IF; reacts with mouse CTCF. Specificity - antibody detects endogenous levels of total CTCF protein; does not cross-react with BORIS.

Anti-CTCF [EPR7314(B)]:
-supplier website: ChIP Grade/suitable for ChIP, reacts with mouse CTCF. Validated for ChIP in HeLa cells for enrichment at known CTCF binding site relative to IgG control and negative control region.
-ChIP-qPCR experiments in this study show strong enrichment at known CTCF binding sites compared to negative control regions (Supplementary Fig. 5i).

Anti-SP1 [ARC0128]:
-supplier website: Verified by Knockdown to ensure that the antibody binds to the antigen stated. Tested for WB and ICC/IF; reacts with mouse.

Anti-SP1 (ab227383):
-supplier website: suitable for WB and ICC/IF; reacts with mouse. Specificity validated by shRNA knock-down.

Anti-YY1 [EPR4652]:
-supplier website: suitable for WB and ICC/IF; reacts with mouse.

Eukaryotic cell lines

Policy information about cell lines and Sex and Gender in Research

Cell line source(s)	Abelson-transformed WT mouse preB cells were previously derived in our lab from transgenic heterozygous Rad21-Tev-Myc mice (Tachibana-Konwalski, Genes Dev, 2010). A female WT line, which was used previously (Djeghloul et al, Nat Comms, 2020), was used for this study.<br>VL3-3M2 (CD4+CD8+) mouse cells are described in (Groves et al, The Journal of Immunology, 1950) and were previously gifted to the lab by Stephen Smale. The sex of this cell line is unknown.<br>J774A.1 is a macrophage cell line isolated in 1968 from the ascites of an adult, female mouse with reticulum cell sarcoma (ATCC, TIB-67).
Authentication	The preB female WT clone was previously validated in the lab by PCR genotyping and western blotting (Djeghloul et al, Nat Comms, 2020) and was karyotyped for this study.<br>Ikaros-mNeonGreen knock-in preB cells derived in this study were validated by western blotting, PCR + sanger sequencing, and comparison of live-cell imaging with antibody staining of endogenous Ikaros, and were karyotyped.<br>PBK KO preB cells were validated by western blot and PCR + sanger sequencing, and were karyotyped.<br>VL3-3M2 and J774A.1 cells were confirmed to show the expected morphology and to express Ikaros.
Mycoplasma contamination	Mouse preB cells tested negative for mycoplasma contamination. Mouse VL3-3M2 and J774A.1 cells were not tested for mycoplasma for this study.
Commonly misidentified lines<br>(See ICLAC register)	No commonly misidentified cell lines were used in this study.

Plants

Seed stocks	*Report on the source of all seed stocks or other plant material used. If applicable, state the seed stock centre and catalogue number. If plant specimens were collected from the field, describe the collection location, date and sampling procedures.*
Novel plant genotypes	*Describe the methods by which all novel plant genotypes were produced. This includes those generated by transgenic approaches, gene editing, chemical/radiation-based mutagenesis and hybridization. For transgenic lines, describe the transformation method, the number of independent lines analyzed and the generation upon which experiments were performed. For gene-edited lines, describe the editor used, the endogenous sequence targeted for editing, the targeting guide RNA sequence (if applicable) and how the editor was applied.*
Authentication	*Describe any authentication procedures for each seed stock used or novel genotype generated. Describe any experiments used to assess the effect of a mutation and, where applicable, how potential secondary effects (e.g. second site T-DNA insertions, mosiacism, off-target gene editing) were examined.*

Flow Cytometry

Plots

Confirm that:

☒ The axis labels state the marker and fluorochrome used (e.g. CD4-FITC).

☒ The axis scales are clearly visible. Include numbers along axes only for bottom left plot of group (a 'group' is an analysis of identical markers).

☒ All plots are contour plots with outliers or pseudocolor plots.

☒ A numerical value for number of cells or percentage (with statistics) is provided.

Methodology

Sample preparation	Mitotic chromosomes:<br>Demecolcine-arrested preB cells (~10^8) were incubated for 20 min at RT in 10 ml of hypotonic solution (75 mM KCl, 10 mM MgSO4, 0.5 mM spermidine trihydrochloride (Sigma-Aldrich, S2501), 0.2 mM spermine tetrahydrochloride (Sigma-Aldrich, S2876), pH 8.0), followed by 15 min on ice in 3 ml of polyamine buffer (80 mM KCl, 15 mM Tris-HCl, 2 mM EDTA, 0.5 mM EGTA, 3 mM DTT, 0.25% Triton X-100, 0.5 mM spermidine trihydrochloride, 0.2 mM spermine tetrahydrochloride, pH 7.7). Samples were vortexed for 30 s at maximum speed, passed several times through a 21-gauge needle, centrifuged at 200 g for 2 min, filtered through a 20 µm CellTrics filter (Sysmex) and stored at 4 °C overnight. Next day, chromosomes were stained on ice by adding 5 µg/ml Hoechst 33258 (Sigma-Aldrich, 94403), 25 µg/ml Chromomycin A3 (Sigma-Aldrich, C2659) and MgSO4 (10 mM final) for 45 min, followed by addition of sodium citrate (10 mM final) and sodium sulphite (25 mM final) for 1 h.<br><br>PI cell cycle analysis:<br>Demecolcine-arrested preB cells (10^6) were fixed with ice-cold 70% ethanol and stored at −20°C until staining. Fixed cells were washed once with PBS and incubated with PI stain (1X PBS, 0.05 mg/ml PI (Sigma-Aldrich, P4864), 1 mg/ml RNase A, 0.05% IGEPAL CA-630) for 10 min at room temperature (RT) and 20 min on ice.<br><br>Mitotic cell sorting (based on PI & H3S10p):<br>Demecolcine-arrested preB cells (50-60x10^6) were crosslinked in 10 ml 1% methanol-free formaldehyde (Thermo Scientific) in PBS (10min, RT), quenched with glycine (Active Motif, 5 min, RT), washed once with PBS, snap-frozen and stored at -80 °C. Fixed cell pellets were thawed on ice, resuspended in 3-5 ml permeabilisation/blocking buffer (1X PBS, 10% normal goat serum, 2 mM EDTA, 0.1% Triton X-100, 1X cOmplete protease inhibitor cocktail (Roche, 11697498001) and incubated at RT for 15 min. Samples were stained by addition of RNase A (1 mg/ml final concentration), PI (0.05 mg/ml final concentration) and Alexa Fluor 488 anti-H3S10p antibody (1:1000, Table 4) and incubating at RT for 20 min, and then kept on ice until FACS.
Instrument	Mitotic chromosomes:<br>Stained chromosomes were purified using a customised Becton Dickinson Influx, using a 70 µm nozzle tip, a drop-drive frequency of 96 kHz and a sheath pressure of 448 kPa. Forward scatter was measured with a 488 nm laser (Coherent Sapphire, 200 mW); Hoechst 33258 was analysed with a 355 nm air-cooled laser (Spectra Physics Vanguard, 350 mW) and 400 nm (long-pass)/500 nm (short-pass) filters; Chromomycin A3 was analysed with a water-cooled 460 nm laser (Coherent Genesis, 500 mW) and 500 nm (long-pass)/600 nm (short-pass) filters.<br><br>PI cell cycle analysis:<br>BD FACSymphony A3 flow cytometer<br><br>H3S10p mitotic cell sorting:<br>BD FACSAria Fusion flow cytometer
Software	Mitotic chromosomes: BD FACS Software (v1.2.0.142)<br>PI cell cycle analysis: BD FACSDiva Software (v9.1)<br>H3S10p mitotic cell sorting: BD FACSDiva Software (v9.4)
Cell population abundance	Gating strategy and percentages of total chromosomes (40-50% of total events) and individual chromosomes 3 and 19 (1.5-2.5% of total events) are provided in the manuscript. Purity of individual chromosome sorting was previously verified as 99-100% by DNA FISH with mouse chromosome-specific paints (Djeghloul et al, Nat Comms, 2020).<br><br>For PI cell cycle analysis, an average of 15-16% of asynchronous cells were in G2/M whilst an average of 42-47% of demecolcine-arrested cells were in G2/M. Gating strategy and percentages are provided in the manuscript.<br><br>For mitotic cell sorting, an average of 14.1-14.5% cells were in the high PI (4N) / high H3S10p population identified as mitotic cells. Re-analysis of sorted cells confirmed >99% were H3S10p positive. Gating strategy and percentages are provided in the manuscript.
Gating strategy	Chromosomes were first gated based on high Hoechst 33258 vs low Forward Scatter Signal to exclude debris and clumps. A chromosome karyotype was created from this population by plotting Hoechst 33258 vs Chromomycin A3 fluorescence, allowing total chromosomes or individual chromosomes 3 or 19 to be identified.<br><br>For PI cell cycle analysis, live-cells and singlets were selected based on FSC-A/SSC-A and SSC-H/SSC-A respectively. G2/M cells were identified based on PI intensity. Gating strategy and percentages are provided in the manuscript.<br><br>For mitotic cell sorting, live-cells and singlets were selected based on FSC-A/FSC-A and FSC-H/FSC-W respectively. Mitotic cells were identified based on PI and H3S10p-AF488 intensity. Gating strategy and percentages are provided in the manuscript.

☒ Tick this box to confirm that a figure exemplifying the gating strategy is provided in the Supplementary Information.
